# High Resolution Analysis of *DMPK* Hypermethylation and Repeat Interruptions in Myotonic Dystrophy Type 1

**DOI:** 10.3390/genes13060970

**Published:** 2022-05-28

**Authors:** Astrid Rasmussen, Mathis Hildonen, John Vissing, Morten Duno, Zeynep Tümer, Ulf Birkedal

**Affiliations:** 1 Kennedy Center, Department of Clinical Genetics, Copenhagen University Hospital, Rigshospitalet, 2600 Glostrup, Denmark; rasmussenastrid4@gmail.com (A.R.); mathis.hildonen@regionh.dk (M.H.); ulf.birkedal@regionh.dk (U.B.); 2 Copenhagen Neuromuscular Center, Department of Neurology, Copenhagen University Hospital, Rigshospitalet, 2100 Copenhagen, Denmark; john.vissing@regionh.dk; 3 Department of Clinical Medicine, Faculty of Health and Medical Sciences, University of Copenhagen, 2200 Copenhagen, Denmark; 4 Department of Clinical Genetics, Copenhagen University Hospital, Rigshospitalet, 2100 Copenhagen, Denmark; morten.dunoe@regionh.dk

**Keywords:** Oxford nanopore, long-read sequencing, DM1, epigenetics, methylation, diagnostics, Cas9

## Abstract

Myotonic dystrophy type 1 (DM1) is a multisystemic neuromuscular disorder caused by the expansion of a CTG repeat in the 3′-UTR of *DMPK*, which is transcribed to a toxic gain-of-function RNA that affects splicing of a range of genes. The expanded repeat is unstable in both germline and somatic cells. The variable age at disease onset and severity of symptoms have been linked to the inherited CTG repeat length, non-CTG interruptions, and methylation levels flanking the repeat. In general, the genetic biomarkers are investigated separately with specific methods, making it tedious to obtain an overall characterisation of the repeat for a given individual. In the present study, we employed Oxford nanopore sequencing in a pilot study to simultaneously determine the repeat lengths, investigate the presence and nature of repeat interruptions, and quantify methylation levels in the regions flanking the CTG-repeats in four patients with DM1. We determined the repeat lengths, and in three patients, we observed interruptions which were not detected using repeat-primed PCR. Interruptions may thus be more common than previously anticipated and should be investigated in larger cohorts. Allele-specific analyses enabled characterisation of aberrant methylation levels specific to the expanded allele, which greatly increased the sensitivity and resolved cases where the methylation levels were ambiguous.

## 1. Introduction

Myotonic dystrophy type 1 (DM1, [OMIM 160900]) is a multisystemic autosomal dominant neuromuscular disorder. Common symptoms include muscular dystrophy, myotonia, fatal cardiac arrhythmias, cognitive impairment, cataracts and endocrine dysfunction [1]. DM1 is one of the most common forms of adult-onset muscular dystrophy estimated to affect 1 in 8000–20,000. The disease severity and age of onset varies from perinatal death to mild symptoms recognised in late adulthood, and the disorder is generally divided into five clinical categories: congenital severe (CDM1), childhood/infantile, juvenile, classical/adult and late-onset mild forms [1,2]. The underlying genetic defect in DM1 is an expansion of a CTG repeat in the 3′ untranslated region (UTR) of the dystrophia myotonica protein kinase gene (*DMPK*), where affected individuals have >50 repeats [3,4]. Transcription of the pathogenic allele results in a toxic gain-of-function mRNA, which leads to global splicing defects by sequestering the splice factor muscle blind-like 1 (MBNL1) and upregulating CUG-binding protein 1 (CUGBP1) [5,6]. The expanded repeat is unstable in both the germline and the somatic cells with a bias for expansion [7,8,9], which results in both increased mosaicism with age [10], and anticipation where disease severity increases, and age of onset decreases in successive generations [2]. 

Interruptions of the CTG repeat with CCG, GGC, CTC or CAG motifs are estimated to occur in 3–11% of DM1 patients [11,12,13,14]. Repeat interruptions are associated with a higher stability of the repeat and thus decreased somatic mosaicism, a milder phenotype, and later age of onset [15,16]. Hypermethylation of the flanking regions of the CTG repeat has previously been reported in patients with DM1, and methylation levels were found to correlate with repeat size, presence of repeat interruptions, earlier onset, and maternal transmission of the pathogenic allele [17,18,19,20]. Furthermore, methylation levels correlated with muscular, respiratory and cognitive functions in individuals with DM1 [20,21], and have been proposed as a more reliable marker of CDM1 than the CTG repeat length [17]. 

In current genetic practice, the presence of the expanded repeat allele is usually investigated with repeat-primed PCR (RP-PCR), and/or Southern blot (SB) hybridization of genomic DNA or long-range PCR products [22,23]. For shorter alleles (up to ~150 repeats), conventional PCR followed by capillary electrophoresis can be applied. The instability of the expanded CTG repeats complicates an estimation of the expanded repeat length, which traditionally has been assessed by SB hybridization, which is a rather tedious analysis, demanding a large amount of DNA. The estimated progenitor allele length (ePAL) representing the transmitted allele is suggested as a valuable marker to differentiate between different clinical categories [24]. ePAL is typically determined by small-pool PCR (SP-PCR) followed by SB hybridization, where the lower boundary of the length distribution is considered as the inherited allele length [25]. Repeat interruptions can either be detected by RP-PCR if the interruptions are located near the 5′ or 3′ end of the repeat [26], or specific interruption sequences can be investigated by enzymatic cleavage of the DNA prior to SB hybridization [20]; however, in routine set up this investigation is not carried out. Similarly, methylation levels which may be a valuable marker of CDM1 are not investigated in routine analysis. 

An ideal set-up for the genetic diagnosis of DM1 and the investigation of prognostic biomarkers would be the development and implementation of a single test, which could simultaneously determine ePAL along with the median size of the expanded repeat allele, detect the presence of repeat interruptions and quantify the methylation levels flanking the expanded repeat. To cover this need, we performed a pilot study employing long read nanopore sequencing of native unamplified DNA (Oxford Nanopore Technologies, Oxford, UK) obtained from four patients with DM1.

## 2. Materials and Methods

### 2.1. Patient Group

DNA extracted from peripheral blood cells of four male patients with maternally inherited, non-congenital DM1 (P1–P4: 16, 43, 23 and 40 years of age at the time of blood sample, respectively) and four age-matched male controls were included in this study. The molecular diagnosis was established using SB of long-range PCR products for P1 and P2 (modal repeat lengths of ~400 and ~600 repeats, respectively), and with repeat primed PCR (RP-PCR) for P3 and P4, each showing an expanded allele of >80 repeats. Bidirectional RP-PCR showed interruptions in the 3′ end of the repeat in P1 but interruptions were not observed in the other patients. DNA methylation levels at 14 sites surrounding the CTG repeat were previously estimated using pyrosequencing [19], and all the patients had high levels of methylation. The project was approved by the National Committee on Health Research Ethics (protocol H-17017556). 

### 2.2. Cas9-Enrichment and Nanopore Sequencing

To investigate repeat expansions, methylation levels and potential repeat interruptions, DNA libraries were prepared with the Cas9 Sequencing Kit (SQKCS9109, Oxford Nanopore Technologies) using the Cas9 guided enrichment technique described by Gilpatrick et al. [27]. To improve sequencing coverage, two Cas9 guide RNAs were used to cleave upstream of the CpG island downstream of *DMPK* targeting the plus (+) strand and downstream targeting the minus (−) strand (target sequences can be found in Supplementary Table S1). Approximately 5 μg genomic DNA was used to prepare libraries and sequencing was carried out using SpotON R9.4.1 flow cells and MinION Mk1B (Oxford Nanopore Technologies, Oxford, UK). The sequencing ran for 72 h and was operated using the MinKNOW software (Oxford Nanopore Technologies). To assess the quality of the Cas9 targeting and sequencing, the total throughput, reads on target, number of reads at the region of interest (ROI), median coverage of ROI, and mean read accuracy was calculated for each sample using the “Cas9 targeted sequencing” workflow from EPI2ME Labs, provided by Oxford Nanopore Technologies (https://labs.epi2me.io/, accessed on 21 June 2021). 

### 2.3. Repeat Length Analysis and Detection of Repeat Interruptions

Guppy (v.4.0.11, downloaded from Oxford Nanopore Technologies) was used for base-calling with the high accuracy model; DNA_r9.4.1_450bps_hac, Minimap2 (v.2.17) [28] was used to align the raw reads to the human reference genome (GRCh38), and STRique (short-tandem repeat identification, quantification and evaluation) was used to analyse repeat expansions [29]. STRique.py count was used along with a file containing information about the repeat and prefix/suffix sequences of 150 bps marking the borders of the repeat to find the number of triplet repeats in the nanopore reads. Results were filtered and repeat lengths of zero were discarded along with the results where the alignment score was less than 3 for control 1 and less than 4 for the other samples. The reads with more than 35 triplet repeats were inspected manually for repeat interruptions, and the percentage of different trinucleotides were calculated using an in-house python script.

### 2.4. Methylation Analysis by Nanopore Sequencing

Three different tools were employed to study methylation levels at the 400 CpG sites in the 4265 bp CpG island surrounding the *DMPK* CTG repeat: Megalodon v.2.3.3 [30] was used with the basecall model res_DNA_r941_min_modbases_5mC_CpG_v001 from Rerio; DeepSignal [31] with model.CpG.R9.4_1D.human_hx1.bn17.sn360.v0.1.7+; and Nanopolish [32]. Nanopolish was used with the suggested cut-off values of log-likelihood > 2.5 for methylated sites and <−2.5 for unmethylated sites, and the methylated fraction was calculated for each site as the number of methylated reads divided by the total number of reads covering that site. An average was calculated from the three tools to achieve a consensus-based methylation pattern upstream and downstream of the CTG-repeat. Nanopolish data were also used to assess the allele-specific methylation after separating reads with the normal or expanded repeat sequence.

### 2.5. Methylation Analysis by MethylationEPIC Array and Pyrosequencing

Besides nanopore sequencing, methylation levels were measured in the four patients and controls using methylation microarrays. Genomic DNA was bisulfite converted and hybridised to Infinium MethylationEPIC arrays (Illumina, San Diego, CA, USA), performed by Eurofins Genomics, Denmark. Quality control was carried out by calculating a detection *p*-value using the R package minfi [33]. Probes with a detection *p* value below 0.01, probes harbouring single nucleotide polymorphisms (SNPs) and probes with known cross-reactivity were excluded from the analysis. Normalisation was performed using quantile normalization. β values for quantification of DNA methylation levels at each CpG site was calculated, and values for the 22 CpG sites within the *DMPK* CpG island were exported for further analysis. Furthermore, previously obtained data from the four patients and controls using bisulfite converted DNA subjected to pyrosequencing [19] were analysed in conjugation with array and nanopore data.

### 2.6. Data Plotting and Method Comparison

R-packages ggplot2 and ggpubr were used to plot all data and calculate Pearson’s correlation test to assess the degree of correlation in methylation levels at the overlapping CpGs between nanopore sequencing, pyrosequencing, and EPIC arrays [34,35]. The method “loess” in ggplot2 was used for smoothed data lines with standard settings. 

## 3. Results

Cas9 targeting and the sequencing passed the quality criteria and the data are presented in Table 1.

### 3.1. Repeat Length

**Figure 1:**
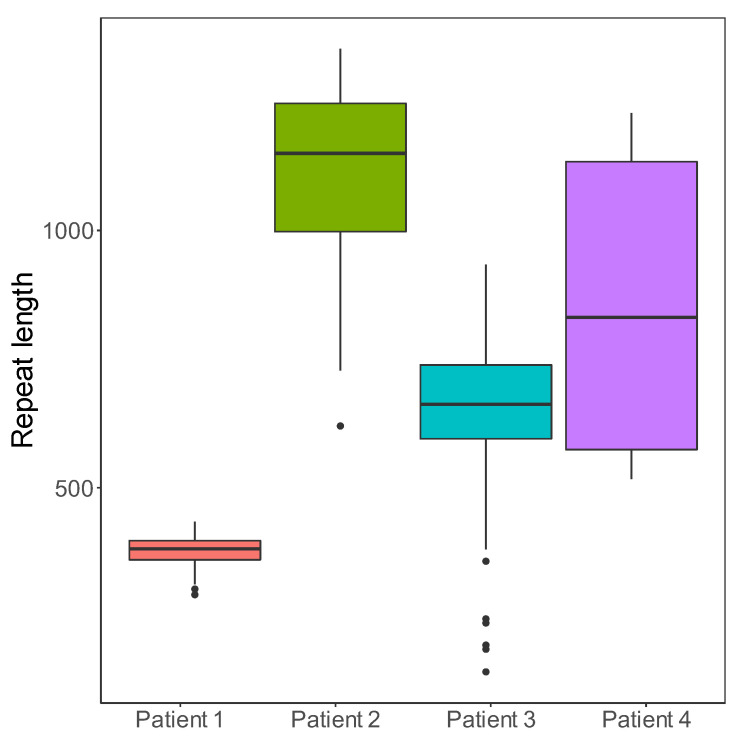
Repeat lengths of the somatically unstable expanded allele for all four patients. The boxplots indicate medians and quartiles of disease allele lengths, and the dots represent outliers.

STRique determined correctly that all the controls had repeat lengths within the normal range (<35). A low degree of variation was observed, which likely represents technical artefacts, owing to the high single nucleotide error rate of nanopore sequencing, causing some minor boundary imprecision from STRique (Supplementary Figure S1). All the patients had one allele within the normal repeat range, and one expanded allele. The expanded allele length showed a high degree of somatic mosaicism (Figure 1, Table 2). The longest individual allele and the longest median repeat length were observed in patient 2, while the shortest individual allele was observed in patient 3. 

### 3.2. Interruptions

**Figure 2:**
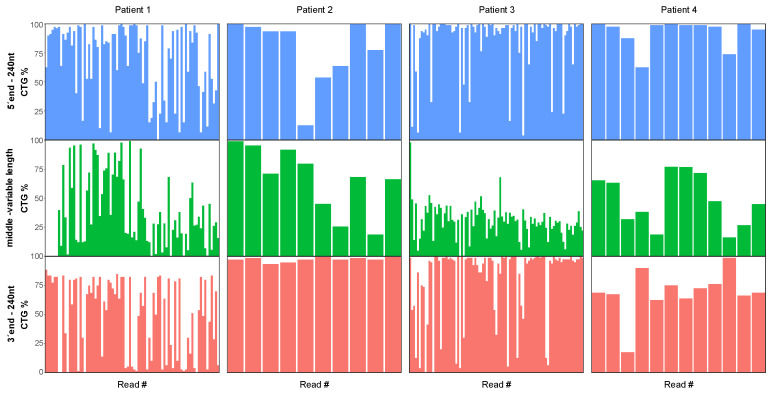
Fraction of CTG triplets per read in the expanded reads of each patient. Each read has been divided into three sections: 240 bp from the 5′ end (blue), the middle part of varying length (green), and 240 bp from the 3′ end (red). The CTG fraction of the reads are lower in the middle part of the repeat, while both the 3′ and 5′ end in three patients (P2, P3 and P4) contains reads that consists entirely of CTG triplets. In patient 1, no reads in the 3′ end of the repeat consist entirely of CTG triplets, which might explain why the interruptions were only observed in the 3′ end of this patient using RP-PCR. The y-axis represents the percentage of CTG trinucleotides in each section of the read sequence, while the x-axis represents the individual reads divided into the 5′ end, the mid-section and the 3′ end.

**Figure 3:**
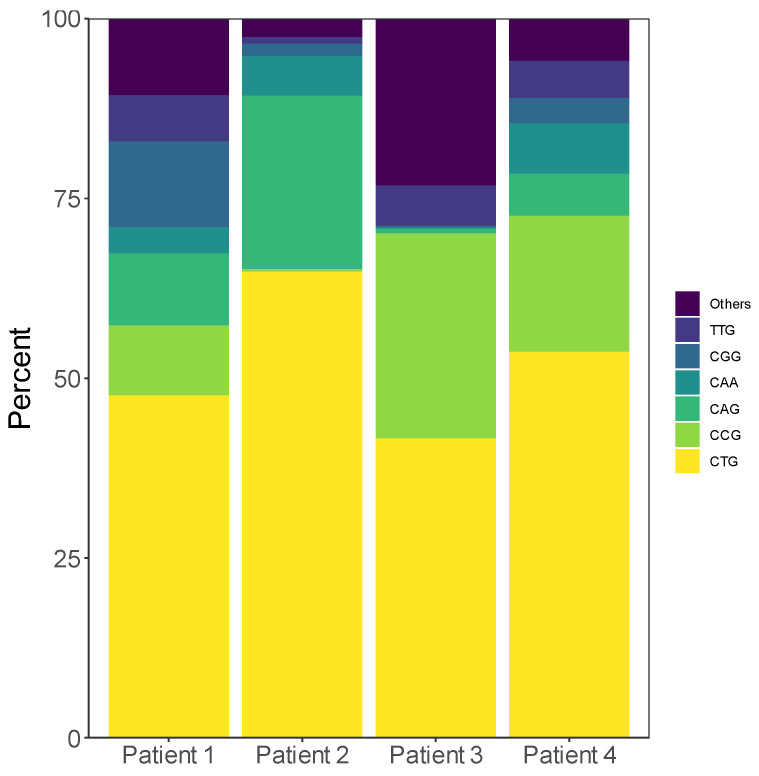
Distribution of the most common triplets in the expanded repeats of each patient.

When inspecting the individual sequence-reads, it became clear that all the patients carried a high degree of repeat interruptions in individual reads, but we were unable to detect a patient-wide pattern or consensus sequence of the interruptions. To assess the data in conjugation with the results from RP-PCR, the expanded sequences identified by STRique were analysed in each read in three sections: 240 nt from the 5′ end, the middle region of varying length, and 240 nt from the 3′ end; 240 nt corresponds to 80 CTG repeats, which is set as the limit for confident detection of interruptions with RP-PCR, and the fraction of CTG trinucleotides were analysed for each section (Figure 2). For all the patients, the middle region of the repeat had a generally lower percentage of CTGs than the ends. The 3′ end of the repeats of P1 differed from the others, as no allele had more than approximately 80% CTG, corresponding to the known interruption. The distribution of the most common trinucleotides in the disease allele and healthy allele of each patient is shown in Figure 3 and Supplementary Figure S2, respectively.

### 3.3. Methylation

**Figure 4:**
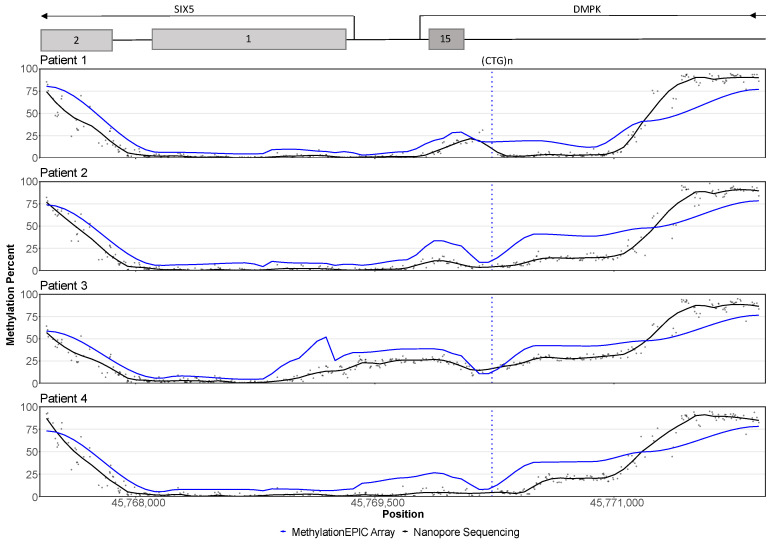
CpG methylation profiles of the patients. EPIC array data (blue lines) from 22 CpG sites is compared to nanopore raw data from 400 CpG sites (black points) and nanopore smoothed data (black lines). The y-axis represents the methylation level in percent and the x-axis represents the genomic position. The position of the CTG repeat is marked with a vertical blue stippled line and the approximate positions of nearby genes are indicated at the top of the plot. Patients 2 and 3 show hypermethylation both upstream and downstream of the repeat. In patient 1, hypermethylation is seen downstream of the repeat, while the methylation levels upstream of the repeat are less clear. Patient 4 has an opposite pattern of patient 1, with clear upstream hypermethylation and possible hypermethylation downstream of the repeat. The methylation patterns observed with EPIC arrays and nanopore sequencing are similar, although nanopore data show lower methylation levels around the repeat, and higher levels towards the upstream end of the CpG island.

The average methylation levels were quantified from both alleles at 400 CpG sites in the CpG island surrounding the *DMPK* CTG repeat. In healthy individuals the CpG island was methylated close to the shores and only a low fraction of methylation was found in the middle of the island (Supplementary Figure S3). Hypermethylation was observed downstream of the repeat in three patients (P1, P2, P3) and hypermethylation upstream of the repeat in three of the patients (P2, P3, P4) (Figure 4). In two individuals, the methylation levels upstream (P1) and downstream (P4) of the repeat could not be clearly characterized as either normal or hypermethylated. Overall, the average methylation levels correlated well with the levels observed by pyrosequencing and EPIC arrays (Supplementary Figure S4), although the nanopore data showed slightly lower levels of hypermethylation (Figure 4). 

**Figure 5:**
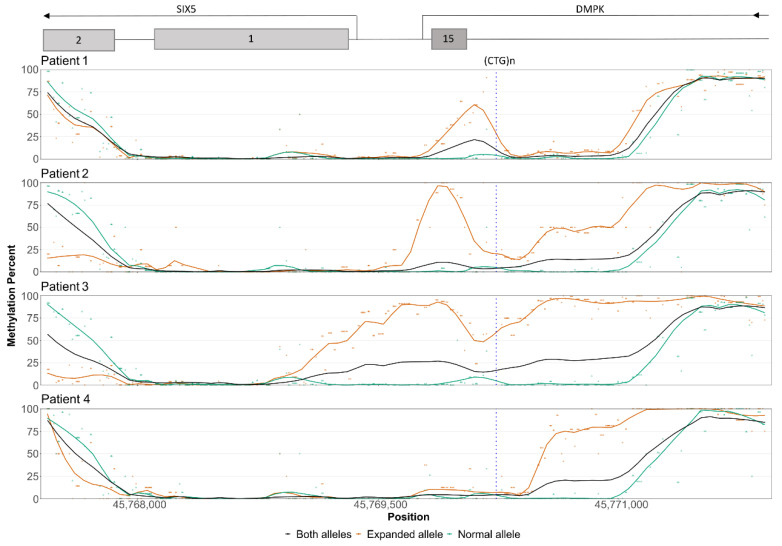
Allele-specific methylation profiles of the patients. The y-axis represents the methylation level in percent and the x-axis represents the genomic position. The vertical blue stippled line indicates the position of the CTG repeat and the positions of nearby genes are indicated at the top of the plot. The average methylation level of all reads is shown by the black line. The healthy alleles (green dots and lines) of the patients display methylation levels similar to the controls. In patient 1, the disease allele (orange dots and lines) is hypermethylated downstream of the repeat, while slightly increased methylation levels upstream of the repeat are also apparent in the disease allele. In patient 4, slight hypermethylation downstream of the repeat can be seen with the allele-specific analysis in addition to the upstream hypermethylation. Patients 2 and 3 have a disease allele that is hypermethylated both upstream and downstream of the repeat. All patients display hypomethylation of the disease allele towards the downstream end of the CpG island. The dots represent individual data points, while the lines are the smoothed data.

Allele-specific methylation analysis revealed that all the patients had a methylation profile comparable to the controls for the normal allele, and a hypermethylated expanded allele (Figure 5). The hypermethylation of the expanded allele was much clearer when allele-specific analysis was employed (Figure 5). Similarly, a slightly lower level of methylation close to the shore downstream of the repeat was observed with both EPIC array and nanopore sequencing, but when investigated in an allele-specific manner, the expanded alleles all showed a profound decrease in methylation levels (Figure 5).

The output from Nanopolish allowed us to analyse the single-read data corresponding to individual native DNA molecules. The hypermethylated areas were examined up- and downstream of the CTG repeat but we were unable to find a correlation between repeat length and methylation density. Methylation data are plotted for individual reads with repeat expansion in Supplementary Figure S5. 

## 4. Discussion

In the present study, we successfully employed Oxford nanopore long read sequencing to simultaneously determine the repeat length, detect repeat interruptions, and quantify methylation levels flanking the expanded *DMPK* CTG repeat in four individuals with DM1 compared to four controls. The available DNA was of varying age and quality, and not extracted for the purpose of long read sequencing, hence some samples resulted in lower coverage than anticipated with the employed Cas9 targeting protocol. However, overall output was satisfying for the aimed analyses.

Nanopore sequencing provided a detailed view of the repeat length mosaicism. As nanopore sequencing provides lengths of the individual alleles, it gives the possibility of estimating the length of the progenitor allele. This may be clinically relevant, as previous studies have suggested that the disease severity has a stronger correlation with the progenitor allele length compared to the modal repeat length [24,36]. For two patients (P1 and P2), original SB results (from 2003 and 2004) were available where the repeat lengths had been estimated to ~400 and ~600 repeats, respectively. Using the same DNA samples, the median repeat length detected with nanopore in P1 (380 repeats) was in line with the SB estimation. In P2, the nanopore results differed from the SB estimations (a median of 1100 repeats vs. ~600 with SB). This discrepancy is likely due to the SB carrying a substantial PCR bias towards shorter fragments, and as P2 had longer repeats than P1, this bias may be more pronounced in this sample. P2 does, however, also have relatively low coverage (10×), and the result is associated with some statistical uncertainty.

Using RP-PCR, we observed interruptions only in P1 [19]. However, using nanopore sequencing, interruptions were detected in all the patients. In three of the patients (P2, P3, P4), the repeat interruptions mainly occurred in the middle of the repeat, with intact stretches of CTG repeats towards each end of the sequence-reads, which explains why it was undetectable with RP-PCR, as RP-PCR can only detect interruptions at the 5′ or 3′ ends. In contrast to our study, where we observed interruptions in all the patients, previous studies have reported 3 to 11% occurrence rate of interruptions when samples were investigated with RP-PCR or enzymatic digestion of PCR products followed by SB [11,12,13]. As nanopore sequencing reveals individual alleles, the method is likely to be more sensitive to detect repeat interruptions, thus interruptions may be more common than anticipated. However, we should underline a selection bias of the patients, as all were selected with the criteria of having high levels of methylation in the regions flanking the repeat, and an association between repeat interruptions and elevated methylation levels have previously been reported [18,19]. Single molecule real-time (SMRT) sequencing by Pacific Biosciences (PacBio) has previously been employed to investigate both the length of expanded alleles and to characterize repeat interruptions, but the methylation levels in the region flanking the repeat were not investigated [37]. Further studies with larger samples sizes are warranted to validate whether repeat interruptions are present in a higher proportion of DM1 patients than previously reported.

Elevated methylation both upstream and downstream of the repeat was detected in all the patients in line with the pyrosequencing and methylation array results, while the controls did not show any methylation. Hypermethylation surrounding the repeat only occurred on the expanded allele, while the normal allele remained unmethylated, which is in line with previous reports [18]. In all patients, allele-specific methylation quantification greatly improved the detection of low-grade hypo- and hypermethylated regions. Allele-specific analysis is preferable for quantifying *DMPK* methylation levels, as it removes the possible influence of the unmethylated normal allele on the results, and hereby provides a higher sensitivity.

The methylation levels detected by nanopore sequencing were significantly correlated with the methylation levels measured by pyrosequencing and EPIC arrays, which is in accordance with a recent large-scale DNA methylation methodology study [38]. However, nanopore data generally indicated lower methylation levels than estimated using pyrosequencing and EPIC arrays. From the repeat length analysis, it was clear that reads from the normal allele were slightly overrepresented, likely due to a bias for shorter sequencing library insert length (Table 2). The average methylation levels would therefore predominantly reflect the normal allele, hence giving rise to the observed differences. In line with this, the observed levels of hypermethylation in the allele-specific analyses is more than two-fold compared to the average levels (Figure 5).

## 5. Conclusions

We have demonstrated that Oxford nanopore sequencing can detect and quantify the length of the expanded *DMPK* CTG repeat in individuals with DM1. As the individual alleles are accurately sequenced and sized, it provides both a detailed view of the somatic instability and allows an estimation of the progenitor allele, which is regarded an important biomarker of disease severity and age of onset. Furthermore, nanopore sequencing can detect and characterise repeat interruptions throughout the entire repeat and provide allele-specific information about the methylation levels surrounding the repeat. The collective expression of all these genetic biomarkers and their conjugative effect on DM1 phenotype is not currently well established. Nanopore sequencing delivers an unprecedented resolution on all of them in a single experiment, making it a powerful tool to understand DM1, and possibly to provide enhanced prognostic information in the future for the benefit of clinicians, patients and family members. SB is no longer a routine analysis in diagnostic laboratories, and long-range sequencing such as nanopore sequencing is undoubtedly a more informative method than RP-PCR. Despite the small sample size, which does not allow biomarker-phenotype correlation, the present study provides a proof-of-concept for the methodology and warrants further studies with larger and more diverse DM1 cohorts, using DNA of high molecular weight.

## Figures and Tables

**Table 1:** Quality control of nanopore sequencing data.

ID	Total Throughput (MB) ^†^	Reads on Target (%) ^‡^	Number of Reads at ROI ^§^	Median Coverage of ROI ^¶^	Mean Read Accuracy (%) ^††^
Patient 1	459.6	1.2	429	215	93.1
Patient 2	202	1.2	159	87	91.9
Patient 3	1000	0.4548	478	212	93.5
Patient 4	224.7	0.1058	69	38	91.2
Control 1	2200	0.0196	136	50	91.8
Control 2	76.8	2.5	107	46	93.2
Control 3	268	0.8184	247	116	93.3
Control 4	421.9	1.3	299	159	93.7

^†^, Total throughput is the amount of data produced; ^‡^, reads on target is the percentage of reads overlapping one of the target sites; ^§^, number of reads at region of interest (ROI) is the number of reads overlapping the *DMPK* target; ^¶^, median coverage of ROI is the average coverage of the *DMPK* target site; ^††^, The accuracy of the read with respect to the reference.

**Table 2:** Age and repeat characteristics of the expanded allele.

ID	Age	Shortest Detected Allele	Median Repeat Length	Longest Detected Allele	Coverage of Expanded Allele	Coverage of Normal Allele
Patient 1	16	292	380	434	80	111
Patient 2	43	620	1100	1353	10	43
Patient 3	23	142	650	934	89	115
Patient 4	40	517	800	1228	12	25

## Data Availability

The data presented in this study are available on reasonable request from the corresponding author.

## Supplementary Materials

The following supporting information can be downloaded at: https://www.mdpi.com/article/10.3390/genes13060970/s1, Supplementary Table S1: Cas9 probes; Supplementary Figure S1: Repeat lengths for all four controls; Supplementary Figure S2: Distribution of most common triplets in the healthy alleles of each patient; Supplementary Figure S3: Nanopore methylation profiles for the controls; Supplementary Figure S4: Correlation analysis; Supplementary Figure S5: Nanopolish data of individual reads.
