# Supplementary material for: High Resolution Analysis of DMPK Hypermethylation and Repeat Interruptions in Myotonic Dystrophy Type 1
Source: Genes (Basel). 2022 May 28;13(6):970. doi: 10.3390/genes13060970 (PMC9222588; doi:10.3390/genes13060970)
Supplement: Supplementary file 1 [file genes-13-00970-s001.zip › genes-1671662-Supplementary.pdf]

Supplementary Table S1: Cas9 probes (Grch38).

**DMPK_5mC_US_1**	ACCCAAGGCTCGCCCAATAG	chr19: 45,763,842-45,763,861	+strand
**DMPK_5mC_US_2**	GGGGAGAGCGGTACCACTTG	chr19: 45,766,586-45,766,605	+strand
**DMPK_5mC_DS_1**	GACGAGGTTACTTCAGACAT	chr19: 45,772,492-45,772,511	-strand
**DMPK_5mC_DS_2**	GGACCTGCGAGTCACACAAC	chr19: 45,783,548-45,783,567	-strand

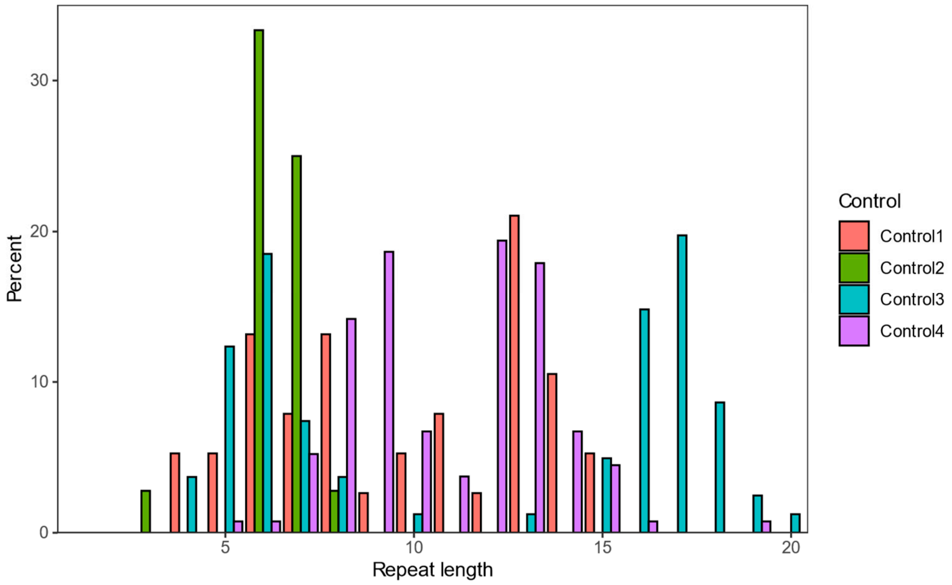

Supplementary Figure S1: Repeat lengths for all four controls.

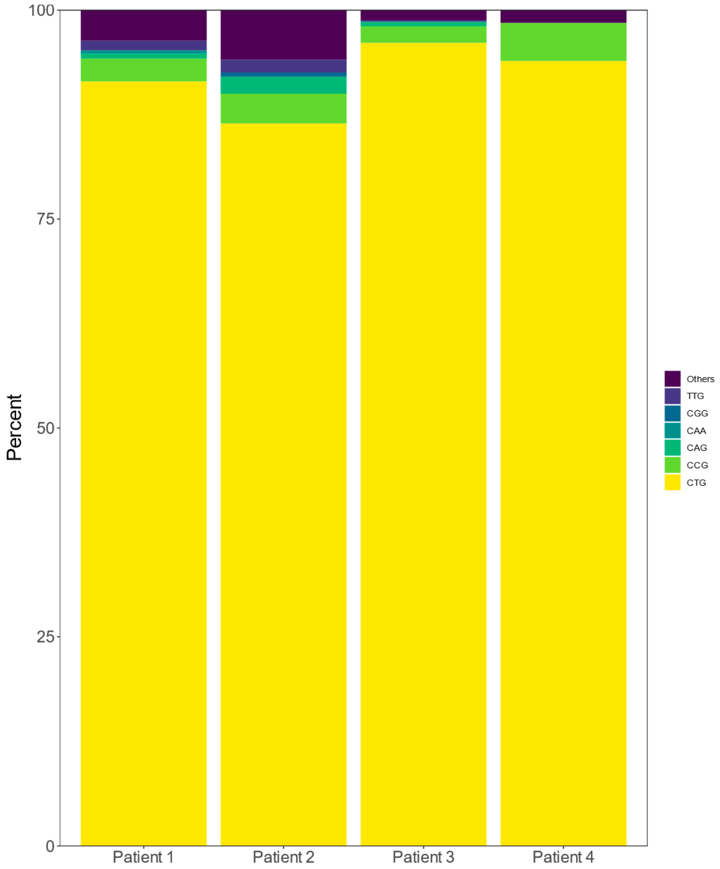

Supplementary Figure S2: *Distribution of most common triplets in the healthy alleles of each patient.*

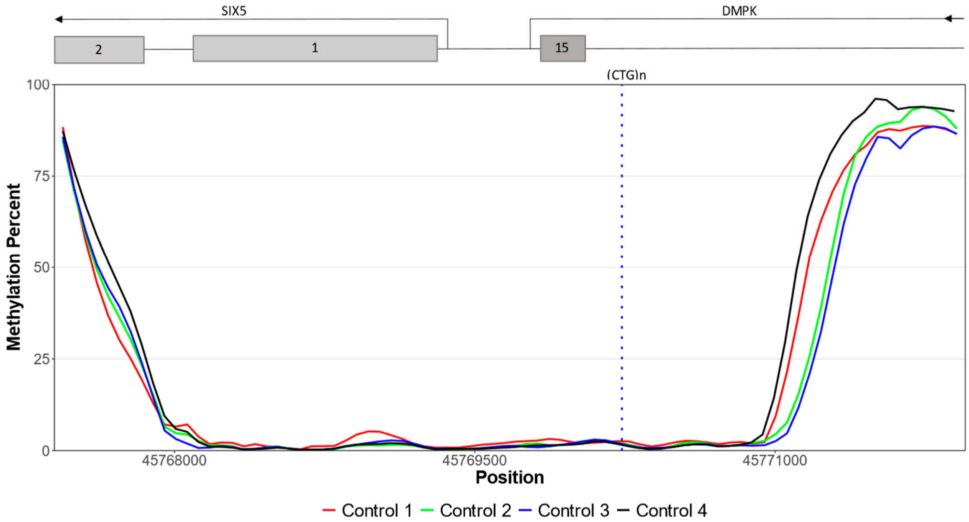

Supplementary Figure S3: Nanopore methylation profiles for the controls. The y-axis represents the methylation level in percent and the x-axis represents the genomic position. The position of the CTG repeat is marked with a blue stippled line and the approximate positions of nearby genes are indicated at the top of the plot.

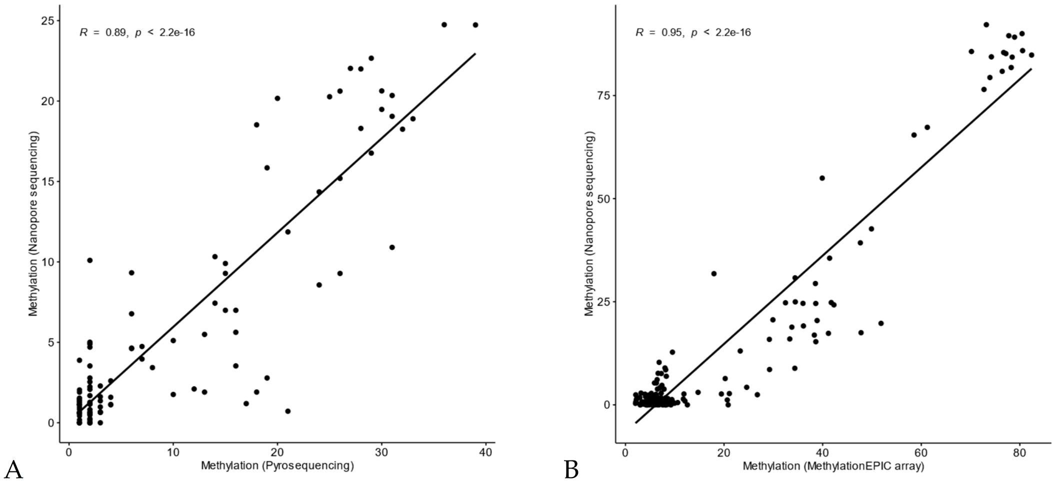

Supplementary Figure S4: Pearson's correlation between methylation levels at the overlapping CpGs between nanopore sequencing and (A) pyrosequencing, and (B) EPIC arrays.

Patient 1 - expanded allele

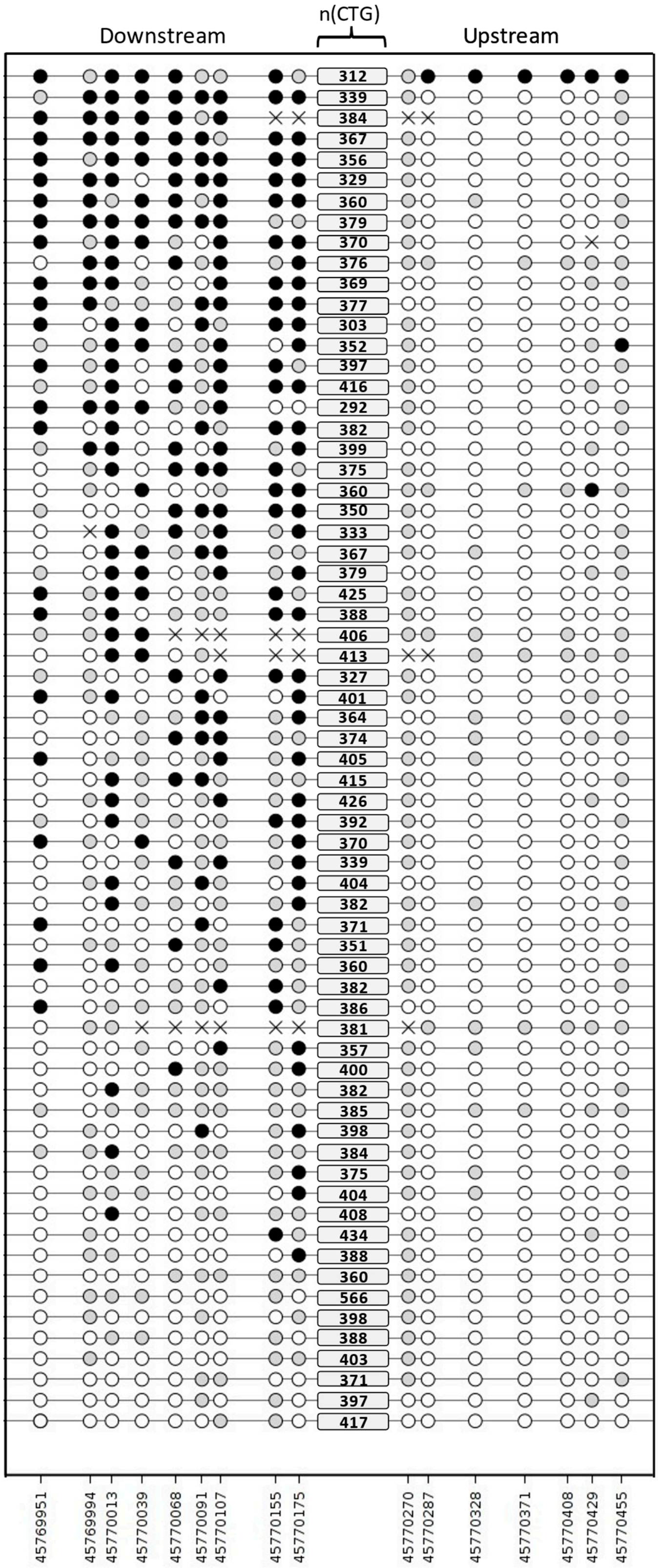

Patient 2 - expanded allele

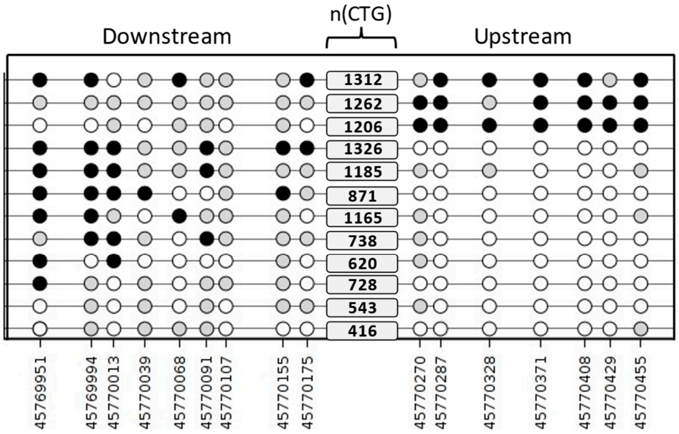

Patient 3 - expanded allele

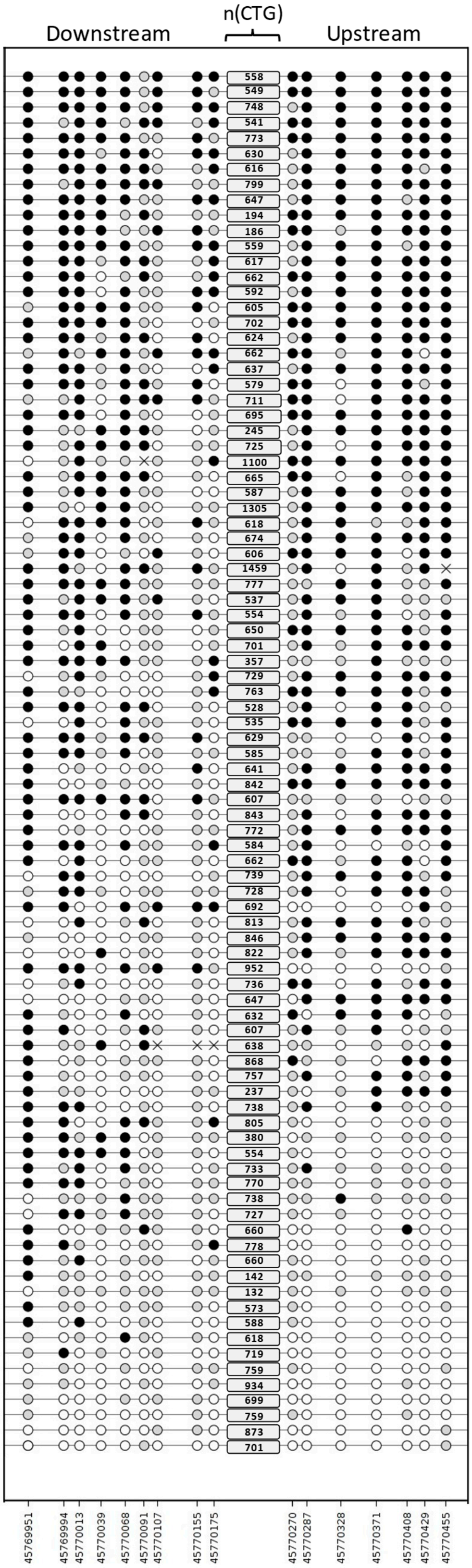

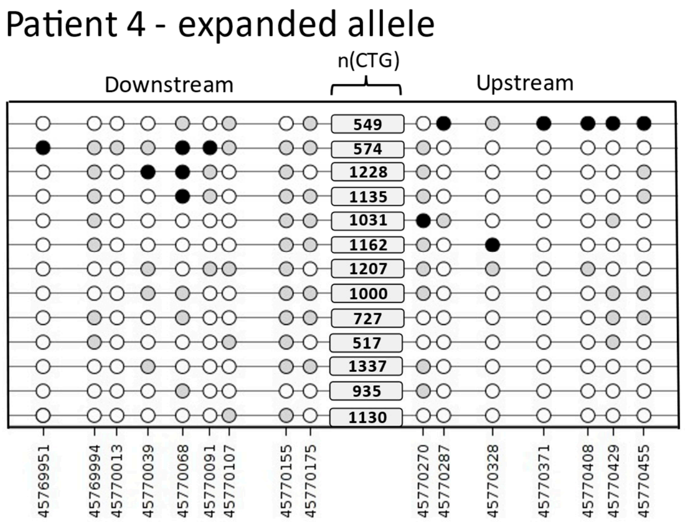

Supplementary Figure S5: Nanopolish data of individual reads from CpG sites in close proximity to the CTG-repeat. Note that adjacent sites are collapsed to yield a single score. Black dots: methylated, white: unmethylated, gray: inconclusive, X: no data.
